# Feasibility and Safety of Flush Endovenous Thermal Ablation of the Great Saphenous Vein with Consecutive Foam Sclerotherapy of Saphenofemoral Junction Tributaries: A Single-Center Experience

**DOI:** 10.3390/jcm13237148

**Published:** 2024-11-26

**Authors:** Jooeun Jun, Myunghee Yoon, Hyukjae Jung, Heejae Jun

**Affiliations:** 1 Department of Vascular Surgery, Jun’s Vascular Clinic, Busan 47256, Republic of Korea; jej142438@gmail.com; 2 Department of Surgery, Pusan National University Hospital, Biomedical Research Institute, Pusan National University, Busan 46241, Republic of Korea; ymh@pusan.ac.kr (M.Y.); goodsight@empas.com (H.J.)

**Keywords:** flush ablation, neovascularization, sclerotherapy, chronic vein insufficiency

## Abstract

**Background:** Blood flow from the saphenofemoral junction(SFJ) tributaries may cause recurrence of varicose veins. Flush occlusion is defined as the total occlusion of the great saphenous vein(GSV) right to the saphenofemoral junction. The purpose of this study was to evaluate the efficacy and safety of flush endovenous thermal ablation with saphenofemoral junction tributary occlusion. **Method:** Between January 2019 and December 2022, 722 patients (total of 1273 limbs) were diagnosed with chronic vein insufficiency by one surgeon at a single center. **Result:** Of the 722 patients, 476 (65.9%) were female and 246 (34.1%) were male. Of the 1273 limbs, endovenous laser ablation(EVLA) was performed in 609 limbs and radiofrequency ablation(RFA) in 664 limbs. Of the 1273 limbs, the recurrence rate was 3.69% (*n* = 47), the development of endovenous heat-induced thromboembolism(EHIT) was 0.31% (*n* = 4), and neovascularization was 1.49% (*n* = 19). **Conclusions:** Flush endovenous thermal ablation was an effective method for decreasing recurrence without inducing endovenous heat-induced thromboembolism. Consecutive foam sclerotherapy for saphenofemoral junction tributaries may be feasible for reducing the recurrence of varicose veins.

## 1. Introduction

Thermal ablation of the great saphenous vein (GSV), including techniques such as endovenous laser ablation (EVLA) and radiofrequency ablation (RFA), has been established as a safe and effective treatment for varicose veins. According to conventional surgical practices, the initiation point for thermal ablation should be 1 to 2 cm distal to the confluence with the superficial epigastric vein (SEV) or the saphenofemoral junction (SFJ) to prevent the progression of thrombosis [1,2,3,4]. However, concerns remain regarding the increased likelihood of recurrence due to residual tributaries at the SFJ [5].

Flush occlusion of the SFJ to the proximal GSV post-thermal ablation has been suggested to yield better long-term outcomes and lower recurrence rates. This is primarily because the flow from cranial tributaries such as the superficial circumflex iliac vein (SCIV), superficial epigastric vein (SEV), and neoreflux in incompetent tributaries like the anterior accessory saphenous vein (AASV) are the most common causes of GSV recurrence after thermal ablation. Simply performing GSV ablation without addressing these tributaries often results in unsatisfactory outcomes.

Therefore, we propose the occlusion of SFJ cranial tributaries such as SCIV, SEV, and AASV during or following flush ablation. This approach aims to comprehensively address all potential sources of reflux and recurrence, thereby enhancing the overall efficacy of the treatment.

The purpose of this study was to evaluate the efficacy and safety of flush endovenous thermal ablation, followed by sclerotherapy of SFJ tributaries. By integrating these techniques, we aim to provide a more robust treatment protocol that minimizes the risk of recurrence and improves patient outcomes. This study seeks to contribute valuable data on the optimization of varicose vein treatments, potentially setting new standards for clinical practice.

## 2. Methods

Between January 2019 and December 2022, spanning a period of 36 months, a total of 722 patients were diagnosed with chronic venous insufficiency by a single surgeon at a single medical center. These patients were treated using flush thermal ablation techniques, specifically endovenous laser ablation (EVLA) and radiofrequency ablation (RFA), encompassing a total of 1273 limbs.

Of the 1273 limbs treated, 609 limbs were subjected to EVLA, with 303 being right limbs and 306 being left limbs. Additionally, 664 limbs were treated with RFA, with 330 being right limbs and 334 being left limbs. Prior to undergoing the procedures, all patients provided written informed consent, ensuring they were fully aware of the treatment process and potential risks involved (Table 1).

A comprehensive evaluation was conducted for each patient, which included taking a full medical history and performing a physical examination. The clinical assessment was thorough and included the Clinical, Etiology, Anatomy, and Pathophysiology (CEAP) classification system to precisely determine the severity and specifics of the venous insufficiency.

### 2.1. Operational Procedure

A venous duplex ultrasound (US) study was performed in all included cases. The US study recorded the compressibility, diameter in centimeters (cm), reflux in seconds for the GSV, and the existence of perforator veins and branch veins on the affected limb.

**Figure 1:**
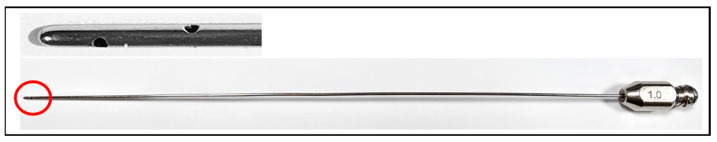
A 20 cm long atraumatic needle (19 G, blunt tip with 3 spiral holes).

All procedures were performed in an ambulatory office-based setting under tumescent local anesthesia and intravenous midazolam. The tumescent local anesthetic solution (2% lidocaine 25 mL and 0.9% saline 500 mL) was infiltrated in the perivenous space under ultrasound guidance using a customized 20 cm long atraumatic needle (19 G, 20 cm, blunt tip with 3 spiral holes) (Figure 1) (MDIF3-10100; Medical Land co., Seoul, Republic of Korea), minimizing the puncture to 3 sites and enabling the separation of the target vein, especially the SFJ with surrounding tissues without vessel injury. Conventionally, 0.5 mg epinephrine is used in tumescent local anesthetic solutions to reduce the bleeding by inducing a vasospasm, but it is unnecessary with a 20 cm long atraumatic needle.

**Figure 2:**
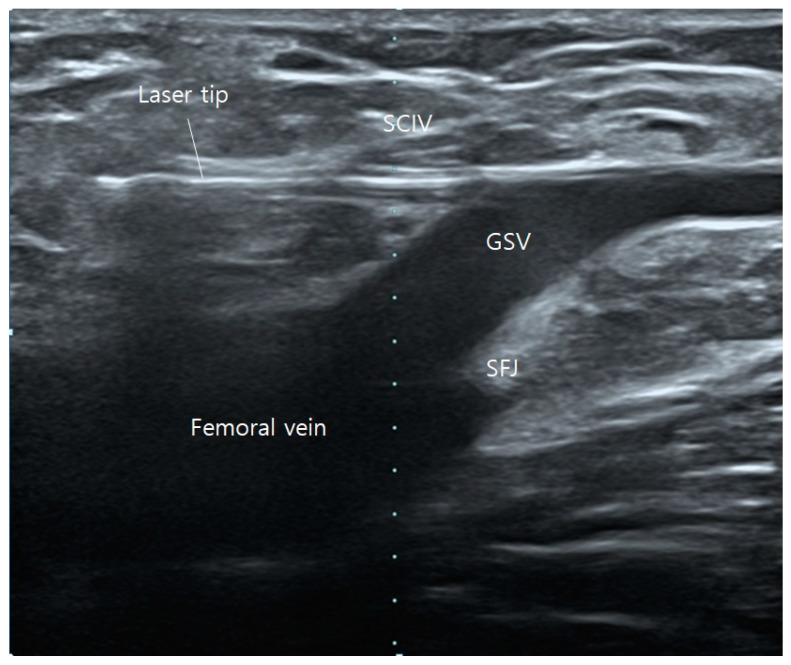
Operative ultrasonography of saphenofemoral junction. Laser tip positioned in superficial circumflex iliac vein (SCIV).

When the superficial epigastric vein (SEV) or the superficial circumflex iliac vein (SCIV) is significantly enlarged with substantial cranial flow causing sacral pain, they are treated concurrently on the day of surgery. Prior to administering tumescent local anesthesia, the EVLA/RFA fiber tip is advanced further into the accessible tributary of either the SEV or SCIV (Figure 2). After the administration of anesthesia, the tributary collapses, making it difficult to advance the tip into the vessel. If the tributaries cannot be accessed during surgery due to vessel angle or small diameter, sclerotherapy can be administered on the day of surgery. This procedure can be performed in upper areas, such as the pelvic region, where tumescent anesthesia is not utilized.

**Figure 3:**
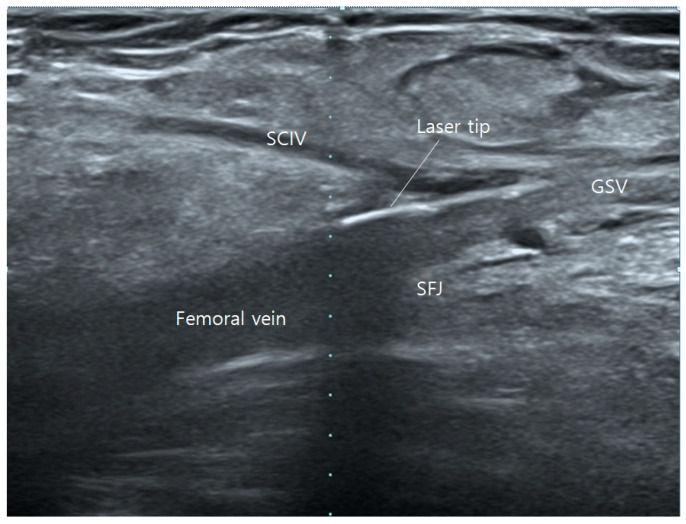
Operative ultrasonography of saphenofemoral junction (SFJ). Juxta-high laser tip placement.

**Figure 4:**
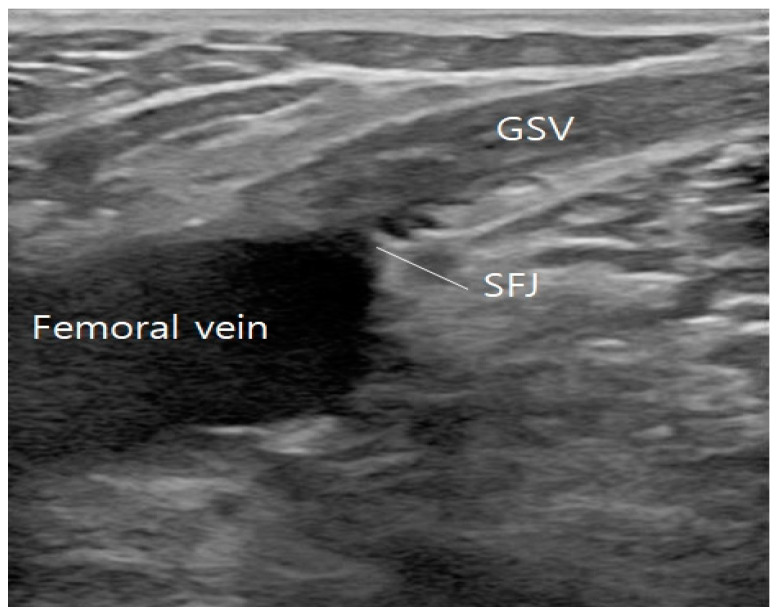
Flush ablation performed on great saphenous vein (GSV) at the saphenofemoral junction (SFJ) and intact femoral vein.

After ablating one of the tributaries, the fiber tip is then brought down to the GSV and moved forward to the SFJ, ablating the GSV until below the knee (Figure 3 and Figure 4), The other left-over tributary observed on ultrasound examination on the next follow-up is treated with foam sclerotherapy.

The EVLA or RFA can be applied only until reaching the knee area to prevent nerve injuries below the knee. But if the vessel below knee is left untreated, it could lead to recurrence via perforating vessels. Therefore, to prevent recurrence, the puncture for the catheter insertion is made in the ankle area, so that after the ablation treatment is performed, the foam sclerotherapy can be applied from below the knee to the ankle while removing the catheter.

### 2.2. Endovenous Laser Ablation (EVLA)

All procedures were performed using a 1940 nm wavelength laser (Atoven; Diotech, Busan, Republic of Korea) with 2.8 French (Fr) ball-type laser fiber. A ball-type laser fiber is safer than a bare tip for reaching the SFJ without causing venous injury in the presence of branches or curves in the blood vessel. A 5 French (Fr) intravenous catheter was placed in the GSV at the ankle area under ultrasound guidance. From January 2019 to March 2020, 4 W laser energy was mainly used, and from April 2020 to December 2021, 7 W was applied. The results of the two groups were analyzed separately. We aimed for a continuous endovenous energy irradiation target of 50–60 J/cm during slow pullback (1 cm every 8 s) of the laser fiber under ultrasound visualization and external compression by the ultrasound probe. The laser energy was administered for a longer time (1 cm every 10 s) at the first 3 cm of the GSV from the SFJ. To minimize deep vein injury due to straight laser heat, the longitudinally positioned ultrasound probe was tilted and compressed proximally to the laser fiber tip at the start of the GSV. The total time and applied energy of the laser treatment were recorded.

### 2.3. Radiofrequency Ablation (RFA)

Radiofrequency energy delivery (Mygen V-700; RF Medical, Seoul, Republic of Korea) was commenced with a temperature of 120 °C using 6 French (F) RFA fiber. Applying compression on the ultrasound probe is important in RFA in order for the blood vessel to contact the heat source. The fiber was then withdrawn 5 cm every 20 s, while double the time of ablation was applied to the first 10 cm from the SFJ.

The first 0.5 cm of the RFA fiber tip does not have a heat-generating coil and does not emit heat in straight direction. When ablating the junction area, less attention is needed with RFA than EVLA, such as tilting the ultrasound probe due to the straight laser heat. As the laser fiber moves through the vessel rather quickly, the RFA fiber remains stationary at a specific point. This allows for more effective heat transfer, ensuring the targeted area receives optimal thermal exposure. Therefore, it is recommended that RFA treatment be performed on the knee area to minimize the risk of nerve injuries below the knee. This precaution is essential because the anatomical structure below the knee is more complex, with nerves that are more susceptible to damage. By limiting the treatment area, practitioners can enhance patient safety while maintaining therapeutic effectiveness.

### 2.4. Foam Sclerotherapy for Tributaries of SFJ

After the operation, ultrasound examination was carried out to check for GSV closure and for any deep vein injuries. If the tributaries of the SFJ were approachable by the fiber during the procedure, it was ablated concomitantly by EVLA or RFA. In cases where they were not accessible, foam sclerotherapy was performed when the superficial epigastric vein (SEV) or the superficial circumflex iliac vein (SCIV) was significantly enlarged with substantial cranial flow causing sacral pain during the regular follow-ups. Ultrasound-guided foam sclerotherapy was performed with sodium tetradecyl sulfate (Fibrovein; STD Pharmaceutical, Hereford, UK) 0.5% combined with room air in a ratio of 1:4 using the Tessari double-syringe system technique. The maximum total volume of foam used per session was <10 mL, in accordance with current guidelines. For patients who underwent sclerotherapy, the sclerosing agent, injection site, volume in milliliters (mL), concentration, and number of injections were recorded.

At our clinic, the first follow-up is scheduled 2–4 weeks after surgery. During this visit, any remaining tributaries are treated. The precise timing of sclerotherapy is not particularly critical; rather, it is essential to occlude all tributaries before neovascularization develops. This ensures that any cranial inflow to the great saphenous vein (GSV) is effectively blocked. Therefore, close observation through doppler ultrasound is necessary to monitor the patient’s progress and ensure successful treatment outcomes.

### 2.5. Postoperative Care

An eccentric compression of the treated veins was applied by using an elastic bandage for 4 h and was replaced with a full-length graduated compression stocking class II (23–32 mmHg) when being discharged. All patients were asked to walk immediately after the procedure.

## 3. Follow-Up

The median follow-up period was 1 year. Patients visited the clinic two weeks, two months, six months, and one year after surgery. An ultrasound study and clinical exam were performed. Patients were asked to document the level of peri- and post-procedural pain and other symptoms. A color duplex scan was performed, scanning the full length of the treated vein, and compressibility and reflux of the vein were tested. A successfully obliterated vein appeared solid with no visible lumen and could not be compressed, showing no flow on color doppler ultrasonography.

Endovenous heat-induced thromboembolism (EHIT) was observed within 2 weeks after surgery. EHIT was treated with Rivaroxaban (Xarelto, Titusville, NJ, USA) 20 mg for 2 months. After the EHIT had resolved, any prescribed medications were promptly discontinued.

## 4. Statistical Analysis

Continuous variables are reported as mean and standard deviation and categorical variables as absolute number and percent, unless stated otherwise. Continuous data were compared using the Student *t*-test for parametric and non-parametric data. Proportion data were compared using Z-tests. Categorical data were compared using the chi-square or Fisher exact tests. Statistical significance was assumed at *p* < 0.05, and the statistical analyses were performed using the language R (http://cran.r-project.org, accessed on 22 December 2022) version 4.2.1. and additional package (stats).

## 5. Results

In a study involving 722 patients, the distribution was 65.9% female (*n* = 476) and 34.1% male (*n* = 246). The total number of limbs treated was 1273. Of these, 609 limbs underwent endovenous laser ablation (EVLA), with 303 right limbs and 306 left limbs treated. Additionally, 664 limbs were treated with radiofrequency ablation (RFA), including 330 right limbs and 334 left limbs. The diameter of the great saphenous vein (GSV) at the saphenofemoral junction averaged 7.42 ± 2.30 mm. According to the Clinical, Etiology, Anatomy, and Pathophysiology (CEAP) classification, 49.87% of patients were classified as C2 (*n* = 360), 42.80% as C3 (*n* = 309), 6.09% as C4 (*n* = 44), 0.69% as C5 (*n* = 5), and 0.55% as C6 (*n* = 4) (Table 2).

Out of the 1273 limbs, the recurrence rate was 3.69% (*n* = 47). The incidence of endothermal heat-induced thrombosis (EHIT) was 0.31% (*n* = 4), and neovascularization occurred in 1.49% of patients (*n* = 19). Specifically, the recurrence rate for EVLA (Group I, 609 limbs) was 5.91% (*n* = 36), compared to 1.65% (*n* = 11) for RFA (Group II, 664 limbs). The development of EHIT was 0.49% (*n* = 3) in the EVLA group and 0.15% (*n* = 1) in the RFA group. Neovascularization rates were 1.31% (*n* = 8) for EVLA and 1.65% (*n* = 11) for RFA (Table 3).

When comparing 4 W EVLA (335 limbs) to 7 W EVLA (274 limbs), the recurrence rate for 4 W was 10.75% (*n* = 36), EHIT development was 0.3% (*n* = 1), and neovascularization was 2.39% (*n* = 8). In contrast, the 7 W EVLA group had a recurrence rate of 0% (*n* = 0), EHIT development of 0.73% (*n* = 2), and neovascularization of 0% (*n* = 0) (Table 4).

Regarding the correlation between varicose vein recurrence and neovascularization of saphenofemoral junction tributaries, among the 36 limbs that recurred after 4 W EVLA, 8 exhibited neovascularization and 28 did not. All 11 limbs that recurred after RFA showed neovascularization (Table 5). This analysis highlights the importance of power settings and procedural techniques in influencing treatment outcomes and complications.

## 6. Discussion

The flush ablation target range was arbitrarily defined as a 0-PD between −2 mm and +1 mm. Distances more than −2 mm were classified as residual stump; distances more than +1 mm were classified as EHIT class 2 to class 4 [6,7,8,9]. With flush thermal ablation performed by one surgeon in our institution, the recurrence rate and the occurrence of EHIT came out to be significantly low. Outcomes after flush thermal ablation treatment were excellent, with no serious complications proving to be an effective method to reduce recurrence and EHIT.

EVLA occludes the vein by inserting the laser fiber into the vessel and irradiating the laser directly into the vessel wall. It has been reported that when the blood is irradiated with a laser, thermal bubbles burn and occlude the vessel wall. As another mechanism, the carbonization of the fiber tip causes the temperature increase inducing collagen contraction of the contacted vessel wall.

Based on our clinical experience, we observed a significantly higher recurrence rate when utilizing a 4 W setting for endovenous laser ablation (EVLA), with a recurrence rate of 10.75%, compared to a 0% recurrence rate observed with a 7 W setting. This marked difference in outcomes led us to transition to the 7 W setting starting in April 2020.

Initially, the laser manufacturing company recommended using the 4 W setting, given that it is a fourth-generation laser with a wavelength of 1940 nm. The company suggested that this configuration would provide sufficient heat transfer to effectively ablate the vessel. However, our practical experience demonstrated that the 4-watt setting was insufficient, resulting in a higher rate of recurrence.

Upon implementing the 7 W setting, we confirmed a substantial improvement in outcomes, with a significantly lower recurrence rate. This finding underscores the importance of selecting the appropriate power setting to ensure optimal thermal ablation efficacy. Consequently, we now recommend the use of a 7 W laser with a wavelength of 1940 nm for EVLA procedures. This adjustment has been shown to provide superior results, effectively reducing the recurrence of varicose veins and enhancing overall treatment success.

Our recommendation is based on empirical evidence and aligns with our goal of achieving the best possible patient outcomes. By adopting the 7 W setting, practitioners can ensure more reliable and effective ablation, thereby minimizing the likelihood of recurrence and improving the long-term success of varicose vein treatments.

Radiofrequency ablation (RFA) is a less invasive technique that uses lower temperatures than laser therapy. It constricts the blood vessel by transmitting the proper temperature (120 °C) using an umbrella-shaped high-frequency catheter, attaching the heating part of the RFA catheter to the vein walls [5,10].

When ablating the junction area, special attention is needed with EVLA, such as tilting due to the straight laser heat. On the other hand, since the 0.5 cm of the RFA fiber tip does not have a heat-generating coil and does not emit heat in straight direction, it is easier for the surgeon to position the fiber tip and can reduce the risk of deep vein injury. RFA was preferred over EVLA for its consistent heat transfer and reduced postoperative complications, such as pain and ecchymosis.

In our institution, all cases of thermal ablation were performed under tumescent local anesthesia as described in the Section 2. Conventionally, 0.5 mg epinephrine is used in tumescent local anesthetic solutions to reduce bleeding by inducing vasospasm, but it is unnecessary with our customized 20 cm long atraumatic needle. Local anesthesia was easily applied on the entire length of the great saphenous vein using a 20 cm long atraumatic needle, making only three puncture sites without damaging the blood vessels. The laser fiber tip was then inserted to a position juxta-high in the SFJ with ultrasound guidance.

During follow-up, some recanalization of the proximal GSV may occur after flush EVLA, but the length of the residual stump and the incidence of reflux in the GSV stump remain significantly lower after flush ablation compared to non-flush ablation [11].

In a study by Shimizu, T et al., the initial success rate of cranial tributary ablation (CTA) was 69%. The AASV occlusion rate (90% vs. 63%, *p* < 0.001) and the flush GSV occlusion rate (68% vs. 30%, *p* < 0.001) at 6 months were better in the CTA group. Their study emphasizes that flush ablation of the GSV at the SFJ significantly improves occlusion rates and reduces the recurrence of varicose veins, particularly by combining cranial tributary ablation (CTA) such as in the AASV [5].

**Figure 5:**
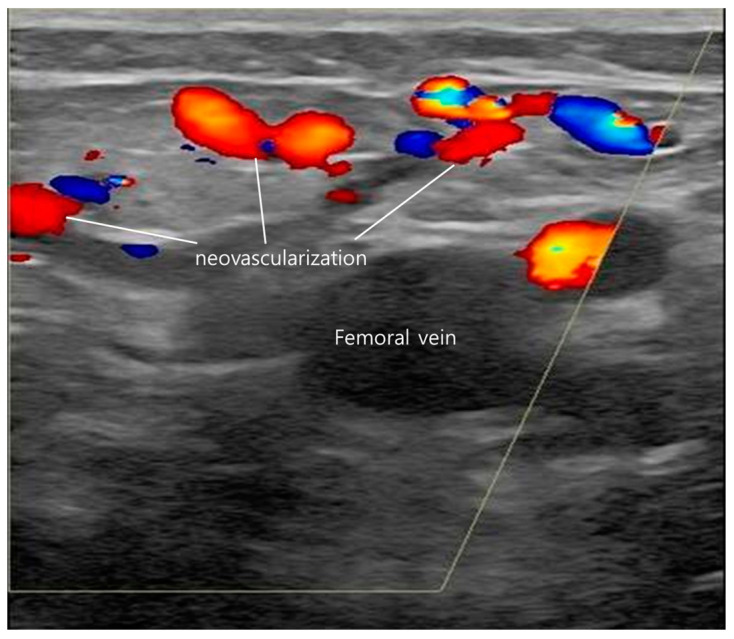
Neovascularization formed in saphenofemoral junction (SFJ) area. Cranial flow appears red in Doppler ultrasonography.

Long-term flush ablation cannot be expected through endovenous thermal ablation alone without complete closure of the cranial tributaries. Even if flush ablation is successful and there is no immediate reflux, if the downward flow of cranial tributaries continues, it can create neovascularization, causing blood flow to proceed downwards, increasing the possibility of recanalization and recurrence of the operated blood vessels. Neovascularization refers to the irregular formation of small collateral vessels in the SFJ area accompanied by reflux (Figure 5). In most cases where neovascularization occurred, the downward flow from the SCIV or SEV was not completely blocked. This is the reason our institution treats the residual tributaries with foam sclerotherapy. Our objective was to completely occlude all inflow to the SFJ region, thereby eliminating any potential for reflux in the GSV.

Regarding the correlation between the recurrence of varicose veins and the neovascularization of SFJ tributaries, of the 47 limbs (4 W EVLA 36 limbs, RFA 11 limbs) recurred, 28 limbs did not have neovascularization, and 19 limbs had neovascularization. Recurrence without neovascularization was additionally treated with revision surgery. The 19 limbs of neovascularization that occurred during the follow-up period were treated with foam sclerotherapy. Foam sclerotherapy is recommended when neovascularization tributaries of the saphenofemoral junction is observed in serial ultrasounds with surveillance [9,11,12,13,14,15].

To reduce the recurrence rate and EHIT, our findings emphasize the significance of precise laser or RFA tip positioning within the SFJ area, along with thorough treatment of the tributaries.

This study has several limitations that should be considered. Firstly, the sample size was relatively small, which may limit the generalizability of our findings. Future research with larger cohorts is necessary to validate these results. Secondly, the follow-up period was limited, potentially affecting the assessment of long-term outcomes and recurrence rates. Extended follow-up studies are needed for a more comprehensive understanding. Additionally, the reliance on a single surgeon’s technique may limit the generalizability of the results. The comparison between different wattages of EVLA was based on observational data, which may introduce bias. The effectiveness of foam sclerotherapy for neovascularization was not explored in a controlled manner. Future research should include larger, randomized trials to validate these findings and explore the long-term efficacy of combined treatment approaches.

## 7. Conclusions

Flush thermal ablation has proven to be an effective method for reducing the recurrence of varicose veins while minimizing the risk of endothermal heat-induced thrombosis (EHIT). This technique provides a targeted approach that enhances treatment efficacy.

Moreover, the use of consecutive foam sclerotherapy for saphenofemoral junction (SFJ) tributaries and neovascularization offers a promising strategy. By effectively blocking any residual or recurrent blood flow into the great saphenous vein, this approach may significantly decrease the likelihood of varicose vein recurrence. The combination of these methods could offer a comprehensive treatment plan that addresses both immediate and long-term patient outcomes.

However, to fully understand the relationship between neovascularization of SFJ tributaries and the recurrence of varicose veins, further research is necessary. Studies involving larger cohorts are needed to provide more definitive evidence on this association. Such research could lead to improved treatment protocols and better management strategies for patients with varicose veins, ultimately enhancing their quality of life and reducing healthcare costs associated with recurrent treatments.

## Figures and Tables

**Table 1:** Overall numbers of treated cases.

	Overall	EVLA	RFA
Patients			
2019	126	126	0
2020	216	133	83
2021	380	94	286
total	722	353	369
Limbs			
2019	216	216	0
2020	373	224	149
2021	684	169	515
total	1273	609	664

**Table 2:** Clinical characteristics of patients between EVLA (Group I) and RFA (Group II).

Variables	Overall (*n* = 722)	Group I (*n* = 353)	Group II (*n* = 369)	*p*-Value
Age (sd)	59.73 (12.61)	62.23 (12.57)	57.34 (12.19)	<0.001
Female/Male (%)	476 (65.9)/246 (34.1)	219 (62.0)/134 (38.0)	257 (69.6)/112 (30.4)	0.038
CEAP classification				
C2	360 (49.87)	171 (48.44)	189 (51.22)	0.379
C3	309 (42.80)	165 (46.75)	144 (39.03)	
C4	44 (6.09)	13 (3.69)	31 (8.40)	
C5	5 (0.69)	2 (0.56)	3 (0.81)	
C6	4 (0.55)	2 (0.56)	2 (0.54)	
Diameter GSV (mm) (sd)	7.42 (2.30)	7.49 (2.37)	7.36 (2.24)	0.308
Limbs (%)				
Left only	89 (12.3)	50 (14.2)	39 (10.6)	-
Right only	82 (11.4)	47 (13.3)	35 (9.5)	-
Both	551 (76.3)	256 (72.5)	295 (79.9)	-
Total (Lt/Rt)	1273 (640/633)	609 (306/303)	664 (334/330)	-

CEAP: Clinical, Etiology, Anatomy, and Pathophysiology.

**Table 3:** Outcomes between EVLA (Group I) and RFA (Group II).

Variables	Overall (1273 Limbs)	Group I (609 Limbs)	Group II (664 Limbs)	*p*-Value
Diameter GSV (mm) (sd)	7.42 (2.30)	7.49 (2.37)	7.36 (2.24)	0.308
Recurrence (%)	47 (3.69)	36 (5.91)	11 (1.65)	<0.01
EHIT (%)	4 (0.31)	3 (0.49)	1 (0.15)	0.138
Neovascularization (%)	19 (1.49)	8 (1.31)	11 (1.65)	0.614

**Table 4:** Outcomes between 4-watt EVLA (Group I) and 7-watt EVLA (Group II).

Variables	Overall (609 Limbs)	Group I (335 Limbs)	Group II (274 Limbs)	*p*-Value
Diameter GSV (mm) (sd)	7.49 (2.37)	7.78 (2.38)	7.15 (2.31)	<0.01
Recurrence (%)	36 (5.91)	36 (10.75)	0 (0)	<0.001
EHIT (%)	3 (0.49)	1 (0.3)	2 (0.73)	0.775
Neovascularization (%)	8 (1.31)	8 (2.39)	0 (0)	<0.001

**Table 5:** Relation between recurrence of varicose veins and neovascularization of the tributaries in 4-watt EVLA (Group I) and 7-watt EVLA (Group II) and RFA (Group III).

Variables	Group I (335 Limbs)	Group II (274 Limbs)	Group III (664 Limbs)	*p*-Value
Recurrence (%)	36 (10.75)	0 (0)	11 (1.65)	<0.001
Neovascularization (%)	8 (2.39)	0 (0)	11 (1.65)	0.047
Recur (+) Neovascularization (+)	8(1.31)	0 (0)	11 (1.65)	0.047
Recur (+) Neovascularization (−)	28(8.35)	0 (0)	0(0)	<0.001
Recur (−) Neovascularization (+)	0 (0)	0 (0)	0 (0)	NaN

NaN: Not a number.

## Data Availability

The data presented in this study are available on request from the corresponding author. The data are not publicly available due to patients’ personal information.
